# QuickStats

**Published:** 2014-08-22

**Authors:** FROM THE NATIONAL CENTER FOR HEALTH STATISTICS

**Figure:**
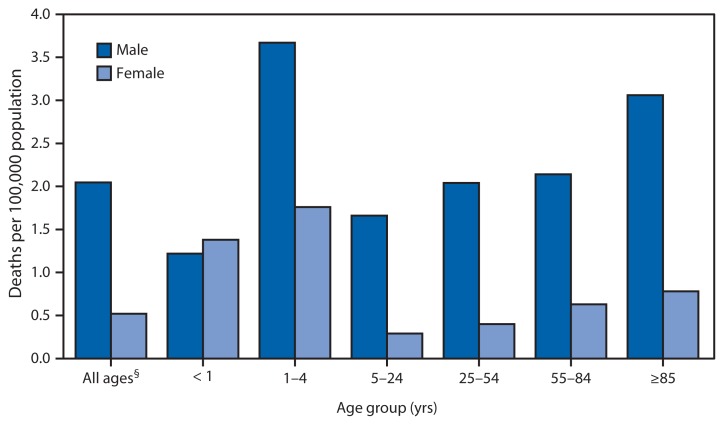
Death Rates from Unintentional Drowning,* by Age Group and Sex — United States,^†^ 2011 * Un intentional drowning as the underlying cause of death includes codes for accidental drowning and submersion (W65–74), watercraft causing drowning and submersion (V90), and water-transport–related drowning and submersion without accident to watercraft (V92) in the *International Classification of Diseases, 10th Revision*. ^†^ U.S. residents only. ^§^ Includes decedents whose ages were not reported.

A total of 3,961 deaths from unintentional drowning were reported in the United States in 2011. In that year, the overall death rate for males was 2.05 per 100,000 population, almost four times the rate for females (0.52). In each age group except for infants (i.e., those aged <1 year), the drowning death rate was higher for males. Males aged 1–4 years had the highest rate (3.67); for males and females, death rates increased with age after age 5–24 years.

**Source:** National Vital Statistics System. Mortality public use data files, 1999–2010. Available at http://www.cdc.gov/nchs/data_access/vitalstatsonline.htm.

**Reported by:** Jiaquan Xu, MD, jiaquanxu@cdc.gov, 301-458-4086.

