# Erratum

**Published:** 2014-09-05

## Vol. 63, No. 33

In the QuickStats, “Death Rates from Unintentional Drowning, by Age Group and Sex — United States, 2011,” an error occurred in the source line. That line should read, “Source: National Vital Statistics System. Mortality public use data file for 2011. Available at http://www.cdc.gov/nchs/data_access/vitalstatsonline.htm.”

